# Editorial: Biochemical and genetic tools to investigate the underlying mechanisms and treatment of sensorimotor pathologies

**DOI:** 10.3389/fnmol.2022.1041458

**Published:** 2022-10-10

**Authors:** Thomas Haynes Hutson, Arnau Hervera

**Affiliations:** ^1^Center for Neuroprosthetics and Brain Mind Institute, Swiss Federal Institute of Technology Lausanne, Lausanne, Switzerland; ^2^Molecular and Cellular Neurobiotechnology, Institute for Bioengineering of Catalonia, Barcelona, Spain; ^3^Department of Cell Biology, Physiology and Immunology, University of Barcelona, Barcelona, Spain; ^4^Network Centre of Biomedical Research of Neurodegenerative Diseases (CIBERNED), Institute of Health Carlos III, Ministry of Economy and Competitiveness, Madrid, Spain; ^5^Institute of Neuroscience, University of Barcelona, Barcelona, Spain

**Keywords:** sensorimotor, neurotrauma and neurodegenerative disease, biochemical engineering, optogenetics and DREADDs, spinal cord injury (SCI)

Sensorimotor integration is key to essential behaviors such as locomotion, feeding and reproduction. These actions require complex, fine-tuned neuronal circuitry in both the spinal cord and brain that integrate sensory information originating from the periphery and motor commands from higher brain centers. However, neurological disorders can cause a significant disruption of these neuronal circuits and therefore deficits in the transmission and integration of these signals resulting in functional impairments and an overall reduction in the quality of life for patients.

Currently, there is a lack of therapies to treat sensorimotor disorders, but recent advances in pharmacological, biochemical and genetic engineering tools have allowed us to investigate and start to unravel the underlying mechanisms that may lead to the identification of novel treatments and improve the sensorimotor deficits caused by neurological disorders.

The manuscripts that comprise this Research Topic provide insights into a range of biochemical engineering strategies to better understand sensorimotor pathologies that occur after spinal cord injury (SCI) and other neurological disorders that may lead to the development of novel treatments.

Boyko et al. investigated the long-term metabolic changes that occur in the brain and spinal cord after a SCI. They uncovered mitochondrial lesions outside of the injury area and a significant downregulation of enzymes, essential for the TCA cycle and amino acid metabolism. These could be partially reversed by administration of thiamine, which acts as a pro-activator of mitochondrial 2-oxoglutarate dehydrogenase complex (OGDHC), the enzyme most affected after SCI, within a clinically viable window, which led to an improvement in locomotor performance. Mechanistically, they also showed how inhibition of OGDHC mimics the brains metabolic profile after SCI. This study provides an example of how understanding molecular mechanisms can lead to the discovery of potential new therapeutic targets and the development of novel treatments (Boyko et al.).

Favicchia et al. identified two pharmacological compounds that fully ameliorate the phenotypical cortical abnormalities associated with the Tbx1 mutant mouse line, which is commonly used as a model of 22q11.2 deletion syndrome. Interestingly, the efficacy of these drugs were not dependent on Tbx1 function or expression, meaning these findings could provide some insights into the pathogenetic mechanisms underlying Tbx1 haploinsufficiency (Favicchia et al.).

Lou et al. compared the efficacy of two Herpes Simplex Virus-Thymidine Kinase inhibitors as selective human cell ablation techniques to assess the efficacy and safety of neural progenitor cell transplants as a therapy after SCI. This study represents a step forward in refining and tailoring cellular therapies for neural traumatic injuries and provides valuable insight into the viability of this treatment strategy (Lou et al.).

Eisdorfer et al. used viral vector mediated chemogenetic activation of sensory neurons in the dorsal root ganglion to dissect their role in promoting hindlimb recovery, assessed using kinematic recordings when in combination with treadmill training after a SCI. Chronic chemogenetic activation, coupled with treadmill training, increased afferent sprouting, and led to some functional improvements. Interestingly, most of these improvements were maintained even after chemogenetic activation was ceased. As highlighted by this study, understanding the functional role that defined subpopulations of sensory neurons play after injury and during rehabilitation, will facilitate the development of improved targeted therapies (Eisdorfer et al.).

Lastly, Ceto and Courtine provide a timely mini review on the currently available optogenetic systems for interrogating defined neuronal populations in the brain and spinal cord, and how to use these tools to better understand their role in circuit reorganization and the recovery of function after neurotrauma. This review highlights how optogenetics can be used to functionally assess the integration of cell grafts with host circuitry and provides a useful guide to which optogenetic strategy will suit different experimental setups with a particular focus on the challenges and limitations associated with implementing these optical systems in damaged CNS tissue (Ceto and Courtine).

The five articles published in this Research Topic provide a diverse insight into the different areas of research aimed at better understanding and correcting sensorimotor deficits caused by neurological disorders. They range from assessing metabolic changes, the efficacy and safety of cell grafts therapies to the latest systems to genetically control neuronal activity in defined neuronal subpopulations.

We hope that this topic will inspire other researchers to leverage and further improve these tools, so that we can continue to unravel the role that each cell population plays during sensorimotor deficits with the aim of fully restoring function to those afflicted by neurological disorders.

## Author contributions

All authors listed have made a substantial, direct, and intellectual contribution to the work and approved it for publication.

## Funding

AH was supported by HDAC3-EAE-SCI Project with ref. PID2020-119769RA-I00 from MCIN/AEI/10.13039/ 501100011033 and Severo Ochoa Unit of Excellence (Institute of Bioengineering of Catalonia) CEX2018-000789-S.

## Conflict of interest

The authors declare that the research was conducted in the absence of any commercial or financial relationships that could be construed as a potential conflict of interest.

## Publisher's note

All claims expressed in this article are solely those of the authors and do not necessarily represent those of their affiliated organizations, or those of the publisher, the editors and the reviewers. Any product that may be evaluated in this article, or claim that may be made by its manufacturer, is not guaranteed or endorsed by the publisher.

